# Brain metastasis DNA methylomes, a novel resource for the identification of biological and clinical features

**DOI:** 10.1038/sdata.2018.245

**Published:** 2018-11-06

**Authors:** Matthew P. Salomon, Javier I. J. Orozco, James S. Wilmott, Parvinder Hothi, Ayla O. Manughian-Peter, Charles S. Cobbs, Richard A. Scolyer, Dave S. B. Hoon, Diego M. Marzese

**Affiliations:** 1 Department of Translational Molecular Medicine, John Wayne Cancer Institute at Providence Saint John’s Health Center, Santa Monica, CA 90404, USA; 2 Melanoma Institute Australia, The University of Sydney, Camperdown, NSW, 2065, Australia; 3 Ben & Catherine Ivy Center for Advanced Brain Tumor Treatment, Swedish Neuroscience Institute, Seattle, WA 98122, USA; 4 Sydney Medical School, The University of Sydney, Camperdown, NSW, 2006, Australia; 5 Royal Prince Alfred Hospital, Sydney, New South Wales, 2050, Australia

**Keywords:** CNS cancer, DNA methylation, Sequencing, Metastasis

## Abstract

Brain metastases (BM) are one the most lethal and poorly managed clinical complications in cancer patients. These secondary tumors represent the most common intracranial neoplasm in adults, most frequently originating from lung cancer, breast cancer, and cutaneous melanoma. In primary brain tumors, such as gliomas, recent advances in DNA methylation profiling have allowed for a comprehensive molecular classification. Such data provide prognostic information, in addition to helping predict patient response to specific systemic therapies. However, epigenetic alterations of metastatic brain tumors with specific biological and translational relevance still require much further exploration. Using the widely employed Illumina Infinium HumanMethylation 450K platform, we have generated a cohort of genome-wide DNA methylomes from ninety-six needle-dissected BM specimens from patients with lung cancer, breast cancer, and cutaneous melanoma with clinical, pathological, and demographic annotations. This resource offers an unprecedented and unique opportunity to identify novel DNA methylation features influencing the behavior of brain metastasis, and thus accelerate the discovery of BM-specific theranostic epigenetic alterations.

## Background & Summary

Patients diagnosed with brain metastasis (BM) have a poor quality of life and a dismal prognosis, with survival ranging from three to 25 months^1,2^. While developments in systemic drug treatments have significantly improved the survival of patients with extracranial metastases, BM lesions have shown limited response rates to these approaches^3^. This trend, along with improvements in neuro-diagnostic imaging techniques, has resulted in an increased incidence of metastatic brain tumors^4^. In large epidemiological studies, 8–10% of patients with solid tumors develop BM, this number increases up to 26% when brain autopsy studies are performed^5–9^. In fact, BM represents the most common intracranial neoplasm in adults, outnumbering even primary brain tumors. Interestingly, reflecting an organotropic behavior of tumor cells, the vast majority of secondary brain neoplasms (75-90%) are originated in patients with lung cancer, breast cancer, and cutaneous melanoma^5–8^.

**Figure 1:**
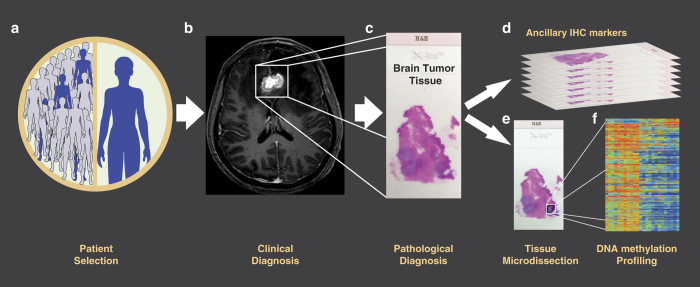
Study design for the construction of genome-wide DNA methylation profiles of metastatic brain tumors. (**a**) Patients with metastatic brain tumors from breast cancer, lung cancer or cutaneous melanoma origin were selected for the study. (**b**) A representative magnetic resonance imaging (MRI) scan of a single metastatic brain tumor lesion used as part of the clinical diagnosis is shown in the scheme. (**c**) After surgery, resected tumors were routinely stored as formalin-fixed and paraffin-embedded (FFPE) tissue blocks and stained with hematoxylin and eosin (H&E) for anatomic pathology diagnosis. (**d**) FFPE tissue sections underwent routine immunohistochemistry (IHC) evaluation to confirm the tumor of origin and molecular subtypes of each case. (**e**) After tumor cell-rich areas were identified, tissue microdissection followed by DNA purification was performed in each case. (**f**) DNA specimens passing the quality control metrics were converted with sodium bisulfite, enzymatically fragmented, and hybridized in the HM450K BeadChips. Raw intensity data were normalized and corrected β values for each specimen were generated. A representative heat map of the DNA methylation data is shown in the study scheme.

Genomic and epigenomic landscapes of primary brain tumors have been extensively investigated. In gliomas, for example, the cytosine-guanine island (CGI) methylator phenotype (CIMP) is frequently found in patients with lower grade gliomas harboring mutations on the *IDH1* gene and is significantly associated with a better overall prognosis^10^. Additially, a favorable response to the DNA alkylating antineoplastic agent, temozolomide, has been directly connected to high DNA methylation (DNAm) level in the promoter region of the *MGMT* gene^11^. DNAm profiling has recently been shown to have the potential to accurately stratify primary central nervous system (CNS) tumours^12^ and to significantly improve the diagnosis of cancer of unknown primary^13^. These clinically relevant findings have demonstrated DNAm profiling to be a valuable tool in the histomolecular evaluation of brain tumors^14,15^. Yet, while genomic and transcriptomic characterization has been performed to some extent^16^, clinically relevant epigenetic alterations of metastatic brain tumors are still poorly understood. Therefore, given this significant knowledge gap, we constructed a comprehensive dataset that can be used to accelerate the identification of novel DNAm features with biological and clinical relevance for the three most frequent types of BM. Here, we present a dataset including genome-wide DNA methylomes constructed using Illumina Infinium HumanMethylation 450K BeadChips (HM450K) of 96 micro-dissected BM specimens from patients with breast cancer, lung cancer, and cutaneous melanoma (Fig. 1). In addition to DNAm data, this report provides a detailed description of the methodological approaches for patient selection, compliance matters, tissue processing and DNA preparation, data normalization, bioinformatics analyses, and usage notes including clinical and demographic information for all patients in the study. Seven of these patients are part of a cohort study that we previously analyzed to identify genome-wide DNAm variations during cutaneous melanoma progression to BM^17–20^. Therefore, the current cohort of BM DNA methylomes is composed of HM450K profiles included in two different NCBI’s Gene Expression Omnibus (GEO) datasets (GSE108576 and GSE44661). We believe that these datasets offer a unique opportunity for the discovery of novel diagnostic and prognostic biomarkers, while simultaneously providing insight into the underlying biology of this serious clinical complication. In this regard, we have employed these data to further explore the utility of DNAm profiles to accurately discriminate between primary and metastatic brain tumors, identify the origin of the BM lesions, and specifically classify BCBM into therapeutically relevant molecular subtypes^21^. Thus, we generated and validated a three-steps BM DNAm based classifier named "BrainMETH"^21^.

## Methods

### Tissue specimen collection

A total of 96 metastatic brain formalin-fixed paraffin-embedded (FFPE) tumor samples from 94 patients diagnosed with breast cancer BM (BCBM; n = 30), lung cancer BM (LCBM; n = 22), and cutaneous melanoma BMs (MBM; n = 44) were included in this study. Two breast cancer patients presented synchronous or asynchronous multiple lesions. The clinical and demographic characteristics of the patients included in the study have been summarized according to relevant information for each cancer type (Table 1). All patient-derived samples and clinical and demographic data were collected under research protocols approved by the joint Institutional Review Board of Providence Saint John’s Health Center/John Wayne Cancer Institute, the Western Institutional Review Board, the Institutional Review Board of Swedish Medical Center, and the Sydney Local Health District (Royal Prince Alfred Hospital Zone) Human Ethics Review Committee. All patients signed an informed consent before joining the study. The experiments were performed in accordance with the World Medical Association Declaration of Helsinki and the National Institutes of Health Belmont Report. Tissues were de-identified and coded according to recommendations of the Health Insurance Portability and Accountability Act (HIPAA) to ensure confidentiality of the patients.

### Histopathological classification of brain metastasis

The BCBM specimens were classified into molecular subtypes according to the expression status of the hormone receptors (HR), i.e. estrogen receptor (ER) and progesterone receptor (PgR), and the human epidermal growth factor receptor 2 (HER2). ER and PgR were assessed by immunohistochemistry (IHC), and HER2 by IHC and/or *in situ* hybridization assays (ISH). FFPE tissue slides were sectioned at 4 μm, mounted onto plus-coated glass slides, and immunohistochemically stained using a Ventana BenchMark ULTRA automated slide stainer (Roche Diagnostics, Indianapolis, IN, USA) by the Clinical Laboratory Improvement Amendments (CLIA)-certified Department of Pathology, Providence Saint John’s Health Center, accredited by the College of American Pathologists (CAP). The antibodies used in this evaluation were the CONFIRM anti-Estrogen Receptor (SP1, #790-4324, Ventana Medical Systems, Tucson, AZ, USA), the CONFIRM anti-Progesterone Receptor (1E2, #790-2223, Ventana Medical Systems, Tucson, AZ, USA), and the PATHWAY anti-HER-2/neu (4B5, #790-2991, Ventana Medical Systems, Tucson, AZ, USA). The scoring criteria for these biomarkers were based on the current ASCO/CAP guidelines^22,23^. Briefly, ER and PgR were considered positive if there was staining of the nucleus in at least ≥1% of tumor cells in the sample. HER2 test result was considered positive if IHC 3+ (observed in a homogeneous and contiguous population and within >10% of the invasive tumor cells) or ISH amplified if single-probe average HER2 copy number >6.0 signals/cell or dual-probe HER2/CEP17 ratio ≥2.0. BCBM specimens were grouped according to the expression of these routinely evaluated markers into three therapeutically relevant subgroups: a- HR positive/HER2 negative, b- HR any/HER2 positive (HER2+), and c- HR negative/HER2 negative (aka triple-negative breast cancer; Table 1).

The MBM samples were categorized according to the mutational status of *BRAF* and *NRAS* genes. Genomic DNA from MBM was amplified with standardized primers specific for exon 15 of *BRAF*, and exons 1 and 2 of *NRAS*^20^. Polymerase chain reaction (PCR) products were purified using QIAquick® PCR Purification Kit (#28106 Qiagen, Germany) and subsequently visualized in 2.2% agarose gel DNA cassettes for gel electrophoresis (FlashGel™ System, Lonza Inc, Rockland, ME, USA). Successfully amplified samples were then quantified by UV absorption spectrophotometry and sequenced using an internal primer^20^ by Eurofins MWG Operon LCC (Eurofins Genomics LCC, Louisville, KY, USA). Sequencing results were analyzed using Chromas Lite v2.6.5 (Technelysium Pty Ltd, Australia) and mutations in *NRAS* and *BRAF* genes were annotated according to the Catalogue of Somatic Mutations in Cancer (COSMIC v86, Wellcome Sanger Institute, Cambridge, UK; http://cancer.sanger.ac.uk/cosmic)^24^. As the presence of *BRAF* and *NRAS* mutations were mutually exclusive events, the MBM specimens were classified into 3 categories: a- BRAF mutated, b- NRAS mutated, and c- BRAF/NRAS wild-type (Table 1). Due to limited tissue availability, two specimens were not profiled for oncogenic mutations on the *BRAF* or *NRAS* genes and presented in Tables 1 and 2 as not available (N/A).

The LCBM were histologically classified into non-small cell lung cancer (NSCLC) and small cell lung cancer (SCLC; Table 1). Of note, we added four BM specimens from female patients with a presumptive diagnosis of LCBM, but with inconclusive IHC analysis, or with a previous diagnosis of both primary lung and breast cancer. In agreement with the clinical-pathological diagnosis an origin of lung cancer was confirmed by DNAm profiling^21^.

### Genomic DNA extraction

Representative FFPE tissue blocks from each metastatic brain lesion were selected by the respective Pathology Departments. FFPE tissue blocks were cut into 4 μm and 8 μm serial slides. Neuropathologists reviewed 4 μm tissue slides stained with hematoxylin & eosin (H&E) for all specimens and labeled representative brain metastatic areas with an estimated tumor purity higher than 70%. After deparaffinization, hematoxylin staining was performed in 8 μm thick serial tissue sections and needle microdissected using the labeled 4 μm tissue slides as template. Genomic DNA (gDNA) was then isolated using ZR FFPE DNA MiniPrep (D3066; Zymo Research, Irvine, CA, USA), according to the manufacturer’s instructions. Genomic DNA was quantified by Qubit® 3.0 Fluorometer (Q33216; Thermo Fisher Scientific, Carlsbad, CA).

### Genome-wide DNA methylation profiling

Sodium bisulfite modification (SBM) was performed on 1 μg of gDNA using the EZ DNA Methylation-Direct Kit (D5021, Zymo Research Irvine, CA, USA). An aliquot of SBM-DNA was analyzed by MethyLight-based quality control to test bisulfite completeness. After correction of SBM-DNA amount, a minimum of 200 ng of SBM-DNA was whole-genome amplified and enzymatically fragmented. Finally, the fragmented SBM-DNA was hybridized into the HM450K (Illumina Inc., San Diego, CA, USA) and scanned using the Illumina iScan microarray scanner following the manufacturer’s recommended settings (Illumina Inc., San Diego, CA, USA).

### Data processing and analysis

**Figure 2:**
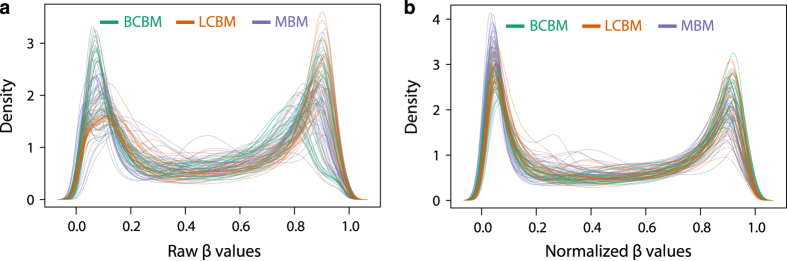
Density distribution of β values across samples. (**a**) The distribution of raw β values for all three BM subtypes before normalization. (**b**) The distribution of β values after normalization for all three BM subtypes. BM subtype is indicated by the color of the line, with green lines representing breast cancer brain metastases (BCBM), orange lines representing lung cancer brain metastases (LCBM), and purple lines representing melanoma brain metastases (MBM).

Data was extracted from Illumina .idat files using the Bioconductor package *minfi*^25^. The ‘*preprocessNoob’* function in *minfi* was used for normalization and dye-bias correction as described in Triche *et al.*^26^. DNAm levels were reported as β-values [β = intensity of the methylated allele/(intensity of the unmethylated allele + intensity of the methylated allele)], and calculated using the signal intensity value for each CpG site. The effect of normalization on the distribution of β values across samples is shown in Fig. 2.

**Figure 3:**
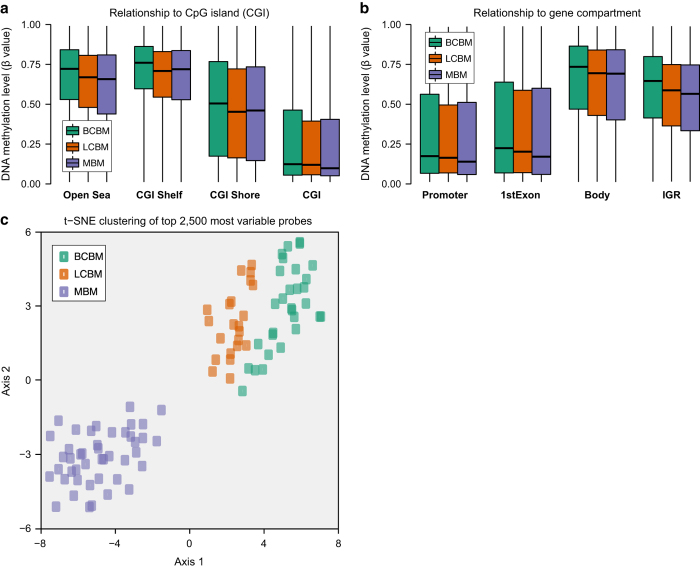
Distribution of β values among genomic features and BM type specific clustering. (**a**) The distribution of DNAm levels in low-CpG density regions (i.e. open sea, CGI shelf, and CGI shore) with respect to high-CpG density CGIs. (**b**) The distribution of DNAm levels for CpG sites with respect to gene compartments in the promoter regions, 5’UTRs, the 1^st^ exon, gene body, and intergenic regions (IGRs). Box colors indicate BM subtype, with green boxes representing breast cancer brain metastases (BCBM), orange boxes representing lung cancer brain metastases (LCBM), and purple boxes representing melanoma brain metastases (MBM). (**c**) t-SNE analysis reveals BM subtype specific clustering of samples. The top 2,500 most variable CpG sites were used for the analysis with the first two dimensions shown. Each of the three BM subtypes is confined to a cluster, with MBMs depicted in purple, BCBMs depicted in green, and LCBMs depicted in orange. No samples were found to fall outside of a BM subtype specific cluster

Using the normalized β values, we compared the genome-wide DNAm profiles for specific genomic features across the three BM groups. DNAm level of CpG sites in high-CpG density regions (known as CpG islands; CGI) and low-CpG density regions (known as CGI shore, CGI shelves, and open sea) were also variable among the three BM groups (Fig. 3a). Additionally, DNAm levels varied among the three BM groups for CpG sites in the promoter regions, 5’UTRs, the first exon, gene body, and intergenic regions (IGRs; Fig. 3b). Finally, to check for overall structure within our dataset, we used the t-distributed stochastic neighbor embedding (t-SNE)^27,28^ method with the top 2,500 most variable HM450K probes to cluster all BM specimens. Three distinct clusters were observed that corresponded to each of the three BM types, with MBMs showing the greatest degree of separation from BCBM and LCBM which were positioned more closely to each other (Fig. 3c). No outlier samples were observed.

### Code availability

All analyses were performed using open source R and Bioconductor packages. Specifically, the *minfi*^25^ package was used to process raw array data and perform normalizations (see “Data processing and analysis” section), summary statistics were calculated using functions in base R and the *matrixStats*^29^ package, density distribution plots were generated using the *densityPlot* function in *minfi*^25^, all other figures were generated using the *ggplot2*^30^ and *RColorBrewer*^31^ packages, and the t-SNE analysis was performed using the *Rtsne*^32^package. No custom code was used in the processing or analysis of this data.

## Data Records

All HM450K raw and normalized data that support the findings of this study have been deposited in the National Center for Biotechnology Information (NCBI) Gene Expression Omnibus (GEO; https://www.ncbi.nlm.nih.gov/geo/) datasets under the series records GSE108576 (Data Citation 1) and GSE44661 (Data Citation 2). The data is presented in a tabular format that includes the unmethylated intensity values, the methylated intensity values, the *p*-value from the statistical evaluation of the differences between signal and noise, and the corrected β value. The DNAm data can also be accessed as raw intensity files (.idat). Additionally, the integration of the clinical-demographic characteristics of the 96 BM specimens with the matched .idat file names and the GEO sample identifiers (GSM) is provided in Table 2 (available online only).

## Technical Validation

**Figure 4:**
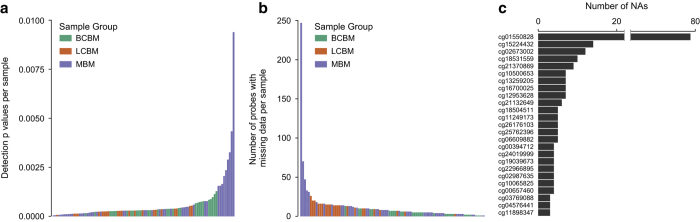
Quality metrics used for sample filtering. A three-part quality control pipeline was used to identify samples with low quality. (**a**) The distribution of mean detection *p*-values for all probes in each sample. (**b**) The distribution of the number of probes with missing data per sample. (**c**) The distribution of the top 25 probes with missing data. For panels **a** and **b**, colors represented BM subtype with green bars representing breast cancer brain metastases (BCBM), orange bars representing lung cancer brain metastases (LCBM), and purple bars representing melanoma brain metastases (MBM).

To ensure that only samples with high overall quality were included in this dataset, we applied a three-step quality control pipeline: 1) We filtered samples by probe detection *p*-value to identify samples with an elevated level of background noise. A significance level of 0.05 for the mean per sample detection *p*-value was used as a cut-off. All 96 samples included in this dataset showed mean detection *p*-values less than 0.05 (Fig. 4a). 2) We calculated the number of probes with missing β values per sample. Across all 96 samples, the median number of probes with missing β values was 7.0 probes per sample with a range of 1 to 246 probes (Fig. 4b). Overall, the number of probes with missing β values represents a minuscule fraction of the total number of probes present on the array and therefore is highly unlikely to have an adverse effect on downstream analysis. 3) For each probe, we calculated the number of samples with missing β values. Notably, of the 485,577 probes included on the HM450K microarray, probe cg01550828 showed missing β values in 79 samples (Fig. 4c). Probe cg01550828 is located in the body of the ring finger protein 168 (*RNF168*) gene and is one of five probes within the *RNF168* gene body. While cg01550828 showed missing values, none of the other four *RNF168* gene body probes showed any missing values across the 96 samples.

## Usage Notes

To enhance the utility of this resource, we have integrated the most relevant clinical and demographic features of the patient cohort and DNAm data for each BM specimen. In Table 2 (available online only), we included patient age at BM diagnosis, gender, primary cancer of origin, and cancer-specific subtypes matched with GEO sample names and .idat identifiers. This information can be accessed from the respective GEO series GSE108576 (Data Citation 1) and GSE44661 (Data Citation 2).

The dataset we present here can be further analyzed to study the differential methylation profiles among the three BM groups described here and/or integrated into larger methylation analyses using new or existing publicly available array data deposited in GEO. Data normalization and differential methylation analysis can be performed using various open source Bioconductor packages. In particular, the ChAMP Bioconductor package provides a comprehensive analysis pipeline that utilizes many well-established methods for the normalization and analysis of Illumina HM450K microarray data^33^. This package is well documented and provides a useful first pass pipeline for processing array data.

## Additional information

**How to cite this article**: Salomon, M. P. *et al.* Brain metastasis DNA methylomes, a novel resource for the identification of biological and clinical features. *Sci. Data*. 5:180245 doi: 10.1038/sdata.2018.245 (2018).

**Publisher’s note**: Springer Nature remains neutral with regard to jurisdictional claims in published maps and institutional affiliations.

## Supplementary Material



## Figures and Tables

**Table 1:** Clinical-demographic characteristics of patients with brain metastasis.

	**BCBM**	**LCBM**	**MBM**	**Total BM**
N	28	22	44	94
**Age (years)**				
Median	54.5	67.5	58.45	61.35
IQR	19.75	10	20.90	19.81
Range	35-79	43-88	32-82	32-88
**Gender**				
Female	28 (100%)	18 (81.8%)	12 (27.27%)	58 (61.7%)
Male	0	4 (18.2%)	32 (72.73%)	36 (38.3%)
**Cancer Specific Subtypes**				
	HR+/HER2-: 12 (42.86%)	NSCLC: 19 (86.4%)	BRAF mut: 19 (43.19%)	
	HR any/HER2+: 11 (39.28%)	SCLC: 3 (13.6%)	NRAS mut: 18 (40.91%)	
	HR-/HER2-: 5 (17.86%)		BRAF/NRAS wt: 5 (11.36%)	
			N/A: 2 (4.54%)	

**Table 2:** ISA-tab formatted table for the access of genome-wide DNA methylation and clinical-demographic data for each patient in the study

**Sample Name**	**Source name**	**Characteristics[organism]**	**Characteristics[organism part]**	**Characteristics[sample type]**	**Method [Protocol 1]**	**Method [Protocol 2]**	**Method [Protocol 3]**	**Method [Protocol 4]**	**Method [Protocol 5]**	**Factor Value [Age at BM diagnosis (years)]**	**Factor Value [Gender]**	**Factor Value [Primary Cancer Origin]**	**Factor Value [BM Cancer Specific Subtypes]**	**Days to Last Follow-up or Death**	**Overall Survival Status**	**Assay Name**	**Raw or Derived Data File [IDAT file 1]**	**Raw or Derived Data File [IDAT file 2]**	**Data Repository**	**Data Record Accession [GEO Serie]**	**Data Record Accession**
BCBM-01	Patient with brain metastasis	Homo sapiens	Brain metastasis	Microdissected formalin-fixed paraffin-embedded tissue	Tissue specimen collection	Histopathological classification of brain metastasis	Genomic DNA extraction	Genome-wide DNA methylation profiling	Data processing and analysis	43	Female	Breast Cancer	HER2 positive/HR any	3179	Dead	Illumina Infinium 450K Human DNA methylation Beadchip	200397860045_R06C02_Grn.idat	200397860045_R06C02_Red.idat	Gene Expression Omnibus	GSE108576	GSM2905348
BCBM-02	Patient with brain metastasis	Homo sapiens	Brain metastasis	Microdissected formalin-fixed paraffin-embedded tissue	Tissue specimen collection	Histopathological classification of brain metastasis	Genomic DNA extraction	Genome-wide DNA methylation profiling	Data processing and analysis	51	Female	Breast Cancer	HER2 positive/HR any	1296	Dead	Illumina Infinium 450K Human DNA methylation Beadchip	200770460177_R01C01_Grn.idat	200770460177_R01C01_Red.idat	Gene Expression Omnibus	GSE108576	GSM2905349
BCBM-03	Patient with brain metastasis	Homo sapiens	Brain metastasis	Microdissected formalin-fixed paraffin-embedded tissue	Tissue specimen collection	Histopathological classification of brain metastasis	Genomic DNA extraction	Genome-wide DNA methylation profiling	Data processing and analysis	52	Female	Breast Cancer	Hormone receptor positive/HER2 negative	6849	Alive	Illumina Infinium 450K Human DNA methylation Beadchip	200123460166_R06C01_Grn.idat	200123460166_R06C01_Red.idat	Gene Expression Omnibus	GSE108576	GSM2905350
BCBM-04	Patient with brain metastasis	Homo sapiens	Brain metastasis	Microdissected formalin-fixed paraffin-embedded tissue	Tissue specimen collection	Histopathological classification of brain metastasis	Genomic DNA extraction	Genome-wide DNA methylation profiling	Data processing and analysis	52	Female	Breast Cancer	Hormone receptor positive/HER2 negative	6849	Alive	Illumina Infinium 450K Human DNA methylation Beadchip	200123460166_R01C02_Grn.idat	200123460166_R01C02_Red.idat	Gene Expression Omnibus	GSE108576	GSM2905351
BCBM-05	Patient with brain metastasis	Homo sapiens	Brain metastasis	Microdissected formalin-fixed paraffin-embedded tissue	Tissue specimen collection	Histopathological classification of brain metastasis	Genomic DNA extraction	Genome-wide DNA methylation profiling	Data processing and analysis	53	Female	Breast Cancer	HER2 positive/HR any	1529	Alive	Illumina Infinium 450K Human DNA methylation Beadchip	200397860045_R02C02_Grn.idat	200397860045_R02C02_Red.idat	Gene Expression Omnibus	GSE108576	GSM2905352
BCBM-06	Patient with brain metastasis	Homo sapiens	Brain metastasis	Microdissected formalin-fixed paraffin-embedded tissue	Tissue specimen collection	Histopathological classification of brain metastasis	Genomic DNA extraction	Genome-wide DNA methylation profiling	Data processing and analysis	62	Female	Breast Cancer	HER2 positive/HR any	5643	Alive	Illumina Infinium 450K Human DNA methylation Beadchip	200770460177_R02C01_Grn.idat	200770460177_R02C01_Red.idat	Gene Expression Omnibus	GSE108576	GSM2905353
BCBM-07	Patient with brain metastasis	Homo sapiens	Brain metastasis	Microdissected formalin-fixed paraffin-embedded tissue	Tissue specimen collection	Histopathological classification of brain metastasis	Genomic DNA extraction	Genome-wide DNA methylation profiling	Data processing and analysis	79	Female	Breast Cancer	Triple Negative Breast Cancer	1328	Dead	Illumina Infinium 450K Human DNA methylation Beadchip	200397860045_R05C02_Grn.idat	200397860045_R05C02_Red.idat	Gene Expression Omnibus	GSE108576	GSM2905354
BCBM-08	Patient with brain metastasis	Homo sapiens	Brain metastasis	Microdissected formalin-fixed paraffin-embedded tissue	Tissue specimen collection	Histopathological classification of brain metastasis	Genomic DNA extraction	Genome-wide DNA methylation profiling	Data processing and analysis	50	Female	Breast Cancer	HER2 positive/HR any	4777	Alive	Illumina Infinium 450K Human DNA methylation Beadchip	200123460166_R02C02_Grn.idat	200123460166_R02C02_Red.idat	Gene Expression Omnibus	GSE108576	GSM2905355
BCBM-09	Patient with brain metastasis	Homo sapiens	Brain metastasis	Microdissected formalin-fixed paraffin-embedded tissue	Tissue specimen collection	Histopathological classification of brain metastasis	Genomic DNA extraction	Genome-wide DNA methylation profiling	Data processing and analysis	73	Female	Breast Cancer	Triple Negative Breast Cancer	18240	Alive	Illumina Infinium 450K Human DNA methylation Beadchip	200123460166_R03C02_Grn.idat	200123460166_R03C02_Red.idat	Gene Expression Omnibus	GSE108576	GSM2905356
BCBM-10	Patient with brain metastasis	Homo sapiens	Brain metastasis	Microdissected formalin-fixed paraffin-embedded tissue	Tissue specimen collection	Histopathological classification of brain metastasis	Genomic DNA extraction	Genome-wide DNA methylation profiling	Data processing and analysis	77	Female	Breast Cancer	Hormone receptor positive/HER2 negative	1324	Dead	Illumina Infinium 450K Human DNA methylation Beadchip	200123460166_R01C01_Grn.idat	200123460166_R01C01_Red.idat	Gene Expression Omnibus	GSE108576	GSM2905357
BCBM-11	Patient with brain metastasis	Homo sapiens	Brain metastasis	Microdissected formalin-fixed paraffin-embedded tissue	Tissue specimen collection	Histopathological classification of brain metastasis	Genomic DNA extraction	Genome-wide DNA methylation profiling	Data processing and analysis	40	Female	Breast Cancer	Triple Negative Breast Cancer	907	Alive	Illumina Infinium 450K Human DNA methylation Beadchip	200123460166_R02C01_Grn.idat	200123460166_R02C01_Red.idat	Gene Expression Omnibus	GSE108576	GSM2905358
BCBM-12	Patient with brain metastasis	Homo sapiens	Brain metastasis	Microdissected formalin-fixed paraffin-embedded tissue	Tissue specimen collection	Histopathological classification of brain metastasis	Genomic DNA extraction	Genome-wide DNA methylation profiling	Data processing and analysis	50	Female	Breast Cancer	HER2 positive/HR any	989	Alive	Illumina Infinium 450K Human DNA methylation Beadchip	200770460041_R02C01_Grn.idat	200770460041_R02C01_Red.idat	Gene Expression Omnibus	GSE108576	GSM2905359
BCBM-13	Patient with brain metastasis	Homo sapiens	Brain metastasis	Microdissected formalin-fixed paraffin-embedded tissue	Tissue specimen collection	Histopathological classification of brain metastasis	Genomic DNA extraction	Genome-wide DNA methylation profiling	Data processing and analysis	56	Female	Breast Cancer	Triple Negative Breast Cancer	3724	Alive	Illumina Infinium 450K Human DNA methylation Beadchip	200123460166_R03C01_Grn.idat	200123460166_R03C01_Red.idat	Gene Expression Omnibus	GSE108576	GSM2905360
BCBM-14	Patient with brain metastasis	Homo sapiens	Brain metastasis	Microdissected formalin-fixed paraffin-embedded tissue	Tissue specimen collection	Histopathological classification of brain metastasis	Genomic DNA extraction	Genome-wide DNA methylation profiling	Data processing and analysis	44	Female	Breast Cancer	Hormone receptor positive/HER2 negative	2075	Alive	Illumina Infinium 450K Human DNA methylation Beadchip	200123460166_R04C01_Grn.idat	200123460166_R04C01_Red.idat	Gene Expression Omnibus	GSE108576	GSM2905361
BCBM-15	Patient with brain metastasis	Homo sapiens	Brain metastasis	Microdissected formalin-fixed paraffin-embedded tissue	Tissue specimen collection	Histopathological classification of brain metastasis	Genomic DNA extraction	Genome-wide DNA methylation profiling	Data processing and analysis	65	Female	Breast Cancer	Hormone receptor positive/HER2 negative	2511	Dead	Illumina Infinium 450K Human DNA methylation Beadchip	200123460166_R05C01_Grn.idat	200123460166_R05C01_Red.idat	Gene Expression Omnibus	GSE108576	GSM2905362
BCBM-16	Patient with brain metastasis	Homo sapiens	Brain metastasis	Microdissected formalin-fixed paraffin-embedded tissue	Tissue specimen collection	Histopathological classification of brain metastasis	Genomic DNA extraction	Genome-wide DNA methylation profiling	Data processing and analysis	72	Female	Breast Cancer	Hormone receptor positive/HER2 negative	2239	Alive	Illumina Infinium 450K Human DNA methylation Beadchip	200770460041_R01C01_Grn.idat	200770460041_R01C01_Red.idat	Gene Expression Omnibus	GSE108576	GSM2905363
BCBM-17	Patient with brain metastasis	Homo sapiens	Brain metastasis	Microdissected formalin-fixed paraffin-embedded tissue	Tissue specimen collection	Histopathological classification of brain metastasis	Genomic DNA extraction	Genome-wide DNA methylation profiling	Data processing and analysis	40	Female	Breast Cancer	Hormone receptor positive/HER2 negative	1303	Alive	Illumina Infinium 450K Human DNA methylation Beadchip	200770460041_R03C01_Grn.idat	200770460041_R03C01_Red.idat	Gene Expression Omnibus	GSE108576	GSM2905364
BCBM-19	Patient with brain metastasis	Homo sapiens	Brain metastasis	Microdissected formalin-fixed paraffin-embedded tissue	Tissue specimen collection	Histopathological classification of brain metastasis	Genomic DNA extraction	Genome-wide DNA methylation profiling	Data processing and analysis	53	Female	Breast Cancer	HER2 positive/HR any	1529	Alive	Illumina Infinium 450K Human DNA methylation Beadchip	200397860045_R03C02_Grn.idat	200397860045_R03C02_Red.idat	Gene Expression Omnibus	GSE108576	GSM2905365
BCBM-20	Patient with brain metastasis	Homo sapiens	Brain metastasis	Microdissected formalin-fixed paraffin-embedded tissue	Tissue specimen collection	Histopathological classification of brain metastasis	Genomic DNA extraction	Genome-wide DNA methylation profiling	Data processing and analysis	57	Female	Breast Cancer	HER2 positive/HR any	1084	Dead	Illumina Infinium 450K Human DNA methylation Beadchip	200770460180_R01C01_Grn.idat	200770460180_R01C01_Red.idat	Gene Expression Omnibus	GSE108576	GSM2905366
BCBM-21	Patient with brain metastasis	Homo sapiens	Brain metastasis	Microdissected formalin-fixed paraffin-embedded tissue	Tissue specimen collection	Histopathological classification of brain metastasis	Genomic DNA extraction	Genome-wide DNA methylation profiling	Data processing and analysis	46	Female	Breast Cancer	Hormone receptor positive/HER2 negative	1905	Alive	Illumina Infinium 450K Human DNA methylation Beadchip	200770460180_R02C01_Grn.idat	200770460180_R02C01_Red.idat	Gene Expression Omnibus	GSE108576	GSM2905367
BCBM-22	Patient with brain metastasis	Homo sapiens	Brain metastasis	Microdissected formalin-fixed paraffin-embedded tissue	Tissue specimen collection	Histopathological classification of brain metastasis	Genomic DNA extraction	Genome-wide DNA methylation profiling	Data processing and analysis	69	Female	Breast Cancer	Hormone receptor positive/HER2 negative	4554	Dead	Illumina Infinium 450K Human DNA methylation Beadchip	200770460180_R03C01_Grn.idat	200770460180_R03C01_Red.idat	Gene Expression Omnibus	GSE108576	GSM2905368
BCBM-23	Patient with brain metastasis	Homo sapiens	Brain metastasis	Microdissected formalin-fixed paraffin-embedded tissue	Tissue specimen collection	Histopathological classification of brain metastasis	Genomic DNA extraction	Genome-wide DNA methylation profiling	Data processing and analysis	46	Female	Breast Cancer	Hormone receptor positive/HER2 negative	4357	Dead	Illumina Infinium 450K Human DNA methylation Beadchip	200770460180_R04C01_Grn.idat	200770460180_R04C01_Red.idat	Gene Expression Omnibus	GSE108576	GSM2905369
BCBM-24	Patient with brain metastasis	Homo sapiens	Brain metastasis	Microdissected formalin-fixed paraffin-embedded tissue	Tissue specimen collection	Histopathological classification of brain metastasis	Genomic DNA extraction	Genome-wide DNA methylation profiling	Data processing and analysis	71	Female	Breast Cancer	HER2 positive/HR any	4737	Alive	Illumina Infinium 450K Human DNA methylation Beadchip	200770460180_R05C01_Grn.idat	200770460180_R05C01_Red.idat	Gene Expression Omnibus	GSE108576	GSM2905370
BCBM-25	Patient with brain metastasis	Homo sapiens	Brain metastasis	Microdissected formalin-fixed paraffin-embedded tissue	Tissue specimen collection	Histopathological classification of brain metastasis	Genomic DNA extraction	Genome-wide DNA methylation profiling	Data processing and analysis	67	Female	Breast Cancer	Hormone receptor positive/HER2 negative	5125	Alive	Illumina Infinium 450K Human DNA methylation Beadchip	200770460180_R06C01_Grn.idat	200770460180_R06C01_Red.idat	Gene Expression Omnibus	GSE108576	GSM2905371
BCBM-26	Patient with brain metastasis	Homo sapiens	Brain metastasis	Microdissected formalin-fixed paraffin-embedded tissue	Tissue specimen collection	Histopathological classification of brain metastasis	Genomic DNA extraction	Genome-wide DNA methylation profiling	Data processing and analysis	57	Female	Breast Cancer	Triple Negative Breast Cancer	1883	Alive	Illumina Infinium 450K Human DNA methylation Beadchip	200770460180_R01C02_Grn.idat	200770460180_R01C02_Red.idat	Gene Expression Omnibus	GSE108576	GSM2905372
BCBM-27	Patient with brain metastasis	Homo sapiens	Brain metastasis	Microdissected formalin-fixed paraffin-embedded tissue	Tissue specimen collection	Histopathological classification of brain metastasis	Genomic DNA extraction	Genome-wide DNA methylation profiling	Data processing and analysis	61	Female	Breast Cancer	Hormone receptor positive/HER2 negative	9131	Dead	Illumina Infinium 450K Human DNA methylation Beadchip	200770460180_R02C02_Grn.idat	200770460180_R02C02_Red.idat	Gene Expression Omnibus	GSE108576	GSM2905373
BCBM-28	Patient with brain metastasis	Homo sapiens	Brain metastasis	Microdissected formalin-fixed paraffin-embedded tissue	Tissue specimen collection	Histopathological classification of brain metastasis	Genomic DNA extraction	Genome-wide DNA methylation profiling	Data processing and analysis	65	Female	Breast Cancer	Hormone receptor positive/HER2 negative	9131	Dead	Illumina Infinium 450K Human DNA methylation Beadchip	200770460180_R03C02_Grn.idat	200770460180_R03C02_Red.idat	Gene Expression Omnibus	GSE108576	GSM2905374
BCBM-31	Patient with brain metastasis	Homo sapiens	Brain metastasis	Microdissected formalin-fixed paraffin-embedded tissue	Tissue specimen collection	Histopathological classification of brain metastasis	Genomic DNA extraction	Genome-wide DNA methylation profiling	Data processing and analysis	35	Female	Breast Cancer	HER2 positive/HR any	N/A	N/A	Illumina Infinium 450K Human DNA methylation Beadchip	200770460180_R06C02_Grn.idat	200770460180_R06C02_Red.idat	Gene Expression Omnibus	GSE108576	GSM2905375
BCBM-32	Patient with brain metastasis	Homo sapiens	Brain metastasis	Microdissected formalin-fixed paraffin-embedded tissue	Tissue specimen collection	Histopathological classification of brain metastasis	Genomic DNA extraction	Genome-wide DNA methylation profiling	Data processing and analysis	45	Female	Breast Cancer	HER2 positive/HR any	N/A	N/A	Illumina Infinium 450K Human DNA methylation Beadchip	201031670094_R04C01_Grn.idat	201031670094_R04C01_Red.idat	Gene Expression Omnibus	GSE108576	GSM2905376
BCBM-33	Patient with brain metastasis	Homo sapiens	Brain metastasis	Microdissected formalin-fixed paraffin-embedded tissue	Tissue specimen collection	Histopathological classification of brain metastasis	Genomic DNA extraction	Genome-wide DNA methylation profiling	Data processing and analysis	40	Female	Breast Cancer	HER2 positive/HR any	935	Alive	Illumina Infinium 450K Human DNA methylation Beadchip	200770460177_R03C01_Grn.idat	200770460177_R03C01_Red.idat	Gene Expression Omnibus	GSE108576	GSM2905377
BM-01	Patient with brain metastasis	Homo sapiens	Brain metastasis	Microdissected formalin-fixed paraffin-embedded tissue	Tissue specimen collection	Histopathological classification of brain metastasis	Genomic DNA extraction	Genome-wide DNA methylation profiling	Data processing and analysis	58	Female	Lung Cancer	Non-Small Cell Lung Cancer	1642	Dead	Illumina Infinium 450K Human DNA methylation Beadchip	200770460180_R04C02_Grn.idat	200770460180_R04C02_Red.idat	Gene Expression Omnibus	GSE108576	GSM2905378
BM-02	Patient with brain metastasis	Homo sapiens	Brain metastasis	Microdissected formalin-fixed paraffin-embedded tissue	Tissue specimen collection	Histopathological classification of brain metastasis	Genomic DNA extraction	Genome-wide DNA methylation profiling	Data processing and analysis	68	Female	Lung Cancer	Non-Small Cell Lung Cancer	N/A	N/A	Illumina Infinium 450K Human DNA methylation Beadchip	200770460180_R05C02_Grn.idat	200770460180_R05C02_Red.idat	Gene Expression Omnibus	GSE108576	GSM2905379
BM-03	Patient with brain metastasis	Homo sapiens	Brain metastasis	Microdissected formalin-fixed paraffin-embedded tissue	Tissue specimen collection	Histopathological classification of brain metastasis	Genomic DNA extraction	Genome-wide DNA methylation profiling	Data processing and analysis	80	Female	Lung Cancer	Non-Small Cell Lung Cancer	1752	Alive	Illumina Infinium 450K Human DNA methylation Beadchip	200866140110_R01C01_Grn.idat	200866140110_R01C01_Red.idat	Gene Expression Omnibus	GSE108576	GSM2905380
BM-04	Patient with brain metastasis	Homo sapiens	Brain metastasis	Microdissected formalin-fixed paraffin-embedded tissue	Tissue specimen collection	Histopathological classification of brain metastasis	Genomic DNA extraction	Genome-wide DNA methylation profiling	Data processing and analysis	55	Female	Lung Cancer	Non-Small Cell Lung Cancer	736	Alive	Illumina Infinium 450K Human DNA methylation Beadchip	200770460177_R03C02_Grn.idat	200770460177_R03C02_Red.idat	Gene Expression Omnibus	GSE108576	GSM2905381
LCBM-01	Patient with brain metastasis	Homo sapiens	Brain metastasis	Microdissected formalin-fixed paraffin-embedded tissue	Tissue specimen collection	Histopathological classification of brain metastasis	Genomic DNA extraction	Genome-wide DNA methylation profiling	Data processing and analysis	62	Female	Lung Cancer	Non-Small Cell Lung Cancer	1551	Dead	Illumina Infinium 450K Human DNA methylation Beadchip	200770460177_R04C01_Grn.idat	200770460177_R04C01_Red.idat	Gene Expression Omnibus	GSE108576	GSM2905382
LCBM-02	Patient with brain metastasis	Homo sapiens	Brain metastasis	Microdissected formalin-fixed paraffin-embedded tissue	Tissue specimen collection	Histopathological classification of brain metastasis	Genomic DNA extraction	Genome-wide DNA methylation profiling	Data processing and analysis	65	Female	Lung Cancer	Non-Small Cell Lung Cancer	1074	Dead	Illumina Infinium 450K Human DNA methylation Beadchip	200770460177_R05C01_Grn.idat	200770460177_R05C01_Red.idat	Gene Expression Omnibus	GSE108576	GSM2905383
LCBM-03	Patient with brain metastasis	Homo sapiens	Brain metastasis	Microdissected formalin-fixed paraffin-embedded tissue	Tissue specimen collection	Histopathological classification of brain metastasis	Genomic DNA extraction	Genome-wide DNA methylation profiling	Data processing and analysis	74	Female	Lung Cancer	Non-Small Cell Lung Cancer	703	Alive	Illumina Infinium 450K Human DNA methylation Beadchip	200770460177_R06C01_Grn.idat	200770460177_R06C01_Red.idat	Gene Expression Omnibus	GSE108576	GSM2905384
LCBM-04	Patient with brain metastasis	Homo sapiens	Brain metastasis	Microdissected formalin-fixed paraffin-embedded tissue	Tissue specimen collection	Histopathological classification of brain metastasis	Genomic DNA extraction	Genome-wide DNA methylation profiling	Data processing and analysis	71	Female	Lung Cancer	Non-Small Cell Lung Cancer	137	Alive	Illumina Infinium 450K Human DNA methylation Beadchip	200770460177_R01C02_Grn.idat	200770460177_R01C02_Red.idat	Gene Expression Omnibus	GSE108576	GSM2905385
LCBM-06	Patient with brain metastasis	Homo sapiens	Brain metastasis	Microdissected formalin-fixed paraffin-embedded tissue	Tissue specimen collection	Histopathological classification of brain metastasis	Genomic DNA extraction	Genome-wide DNA methylation profiling	Data processing and analysis	67	Female	Lung Cancer	Non-Small Cell Lung Cancer	328	Alive	Illumina Infinium 450K Human DNA methylation Beadchip	200770460177_R02C02_Grn.idat	200770460177_R02C02_Red.idat	Gene Expression Omnibus	GSE108576	GSM2905386
LCBM-08	Patient with brain metastasis	Homo sapiens	Brain metastasis	Microdissected formalin-fixed paraffin-embedded tissue	Tissue specimen collection	Histopathological classification of brain metastasis	Genomic DNA extraction	Genome-wide DNA methylation profiling	Data processing and analysis	66	Female	Lung Cancer	Non-Small Cell Lung Cancer	18	Dead	Illumina Infinium 450K Human DNA methylation Beadchip	200770460177_R04C02_Grn.idat	200770460177_R04C02_Red.idat	Gene Expression Omnibus	GSE108576	GSM2905387
LCBM-09	Patient with brain metastasis	Homo sapiens	Brain metastasis	Microdissected formalin-fixed paraffin-embedded tissue	Tissue specimen collection	Histopathological classification of brain metastasis	Genomic DNA extraction	Genome-wide DNA methylation profiling	Data processing and analysis	63	Female	Lung Cancer	Non-Small Cell Lung Cancer	1146	Alive	Illumina Infinium 450K Human DNA methylation Beadchip	200770460177_R05C02_Grn.idat	200770460177_R05C02_Red.idat	Gene Expression Omnibus	GSE108576	GSM2905388
LCBM-10	Patient with brain metastasis	Homo sapiens	Brain metastasis	Microdissected formalin-fixed paraffin-embedded tissue	Tissue specimen collection	Histopathological classification of brain metastasis	Genomic DNA extraction	Genome-wide DNA methylation profiling	Data processing and analysis	71	Female	Lung Cancer	Small Cell Lung Cancer	583	Alive	Illumina Infinium 450K Human DNA methylation Beadchip	200770460177_R06C02_Grn.idat	200770460177_R06C02_Red.idat	Gene Expression Omnibus	GSE108576	GSM2905389
LCBM-12	Patient with brain metastasis	Homo sapiens	Brain metastasis	Microdissected formalin-fixed paraffin-embedded tissue	Tissue specimen collection	Histopathological classification of brain metastasis	Genomic DNA extraction	Genome-wide DNA methylation profiling	Data processing and analysis	86	Female	Lung Cancer	Non-Small Cell Lung Cancer	360	Dead	Illumina Infinium 450K Human DNA methylation Beadchip	200866140110_R02C01_Grn.idat	200866140110_R02C01_Red.idat	Gene Expression Omnibus	GSE108576	GSM2905390
LCBM-14	Patient with brain metastasis	Homo sapiens	Brain metastasis	Microdissected formalin-fixed paraffin-embedded tissue	Tissue specimen collection	Histopathological classification of brain metastasis	Genomic DNA extraction	Genome-wide DNA methylation profiling	Data processing and analysis	67	Male	Lung Cancer	Small Cell Lung Cancer	1050	Alive	Illumina Infinium 450K Human DNA methylation Beadchip	200866140110_R03C01_Grn.idat	200866140110_R03C01_Red.idat	Gene Expression Omnibus	GSE108576	GSM2905391
LCBM-15	Patient with brain metastasis	Homo sapiens	Brain metastasis	Microdissected formalin-fixed paraffin-embedded tissue	Tissue specimen collection	Histopathological classification of brain metastasis	Genomic DNA extraction	Genome-wide DNA methylation profiling	Data processing and analysis	88	Male	Lung Cancer	Non-Small Cell Lung Cancer	623	Dead	Illumina Infinium 450K Human DNA methylation Beadchip	200866140110_R04C01_Grn.idat	200866140110_R04C01_Red.idat	Gene Expression Omnibus	GSE108576	GSM2905392
LCBM-16	Patient with brain metastasis	Homo sapiens	Brain metastasis	Microdissected formalin-fixed paraffin-embedded tissue	Tissue specimen collection	Histopathological classification of brain metastasis	Genomic DNA extraction	Genome-wide DNA methylation profiling	Data processing and analysis	69	Female	Lung Cancer	Non-Small Cell Lung Cancer	519	Alive	Illumina Infinium 450K Human DNA methylation Beadchip	200866140110_R05C01_Grn.idat	200866140110_R05C01_Red.idat	Gene Expression Omnibus	GSE108576	GSM2905393
LCBM-17	Patient with brain metastasis	Homo sapiens	Brain metastasis	Microdissected formalin-fixed paraffin-embedded tissue	Tissue specimen collection	Histopathological classification of brain metastasis	Genomic DNA extraction	Genome-wide DNA methylation profiling	Data processing and analysis	64	Female	Lung Cancer	Non-Small Cell Lung Cancer	129	Dead	Illumina Infinium 450K Human DNA methylation Beadchip	200866140110_R06C01_Grn.idat	200866140110_R06C01_Red.idat	Gene Expression Omnibus	GSE108576	GSM2905394
LCBM-18	Patient with brain metastasis	Homo sapiens	Brain metastasis	Microdissected formalin-fixed paraffin-embedded tissue	Tissue specimen collection	Histopathological classification of brain metastasis	Genomic DNA extraction	Genome-wide DNA methylation profiling	Data processing and analysis	61	Male	Lung Cancer	Non-Small Cell Lung Cancer	277	Dead	Illumina Infinium 450K Human DNA methylation Beadchip	200866140110_R01C02_Grn.idat	200866140110_R01C02_Red.idat	Gene Expression Omnibus	GSE108576	GSM2905395
LCBM-19	Patient with brain metastasis	Homo sapiens	Brain metastasis	Microdissected formalin-fixed paraffin-embedded tissue	Tissue specimen collection	Histopathological classification of brain metastasis	Genomic DNA extraction	Genome-wide DNA methylation profiling	Data processing and analysis	83	Female	Lung Cancer	Non-Small Cell Lung Cancer	975	Dead	Illumina Infinium 450K Human DNA methylation Beadchip	200866140110_R02C02_Grn.idat	200866140110_R02C02_Red.idat	Gene Expression Omnibus	GSE108576	GSM2905396
LCBM-20	Patient with brain metastasis	Homo sapiens	Brain metastasis	Microdissected formalin-fixed paraffin-embedded tissue	Tissue specimen collection	Histopathological classification of brain metastasis	Genomic DNA extraction	Genome-wide DNA methylation profiling	Data processing and analysis	43	Male	Lung Cancer	Small Cell Lung Cancer	315	Alive	Illumina Infinium 450K Human DNA methylation Beadchip	200866140110_R03C02_Grn.idat	200866140110_R03C02_Red.idat	Gene Expression Omnibus	GSE108576	GSM2905397
LCBM-21	Patient with brain metastasis	Homo sapiens	Brain metastasis	Microdissected formalin-fixed paraffin-embedded tissue	Tissue specimen collection	Histopathological classification of brain metastasis	Genomic DNA extraction	Genome-wide DNA methylation profiling	Data processing and analysis	76	Female	Lung Cancer	Non-Small Cell Lung Cancer	66	Dead	Illumina Infinium 450K Human DNA methylation Beadchip	200866140110_R04C02_Grn.idat	200866140110_R04C02_Red.idat	Gene Expression Omnibus	GSE108576	GSM2905398
LCBM-22	Patient with brain metastasis	Homo sapiens	Brain metastasis	Microdissected formalin-fixed paraffin-embedded tissue	Tissue specimen collection	Histopathological classification of brain metastasis	Genomic DNA extraction	Genome-wide DNA methylation profiling	Data processing and analysis	68	Female	Lung Cancer	Non-Small Cell Lung Cancer	11	Alive	Illumina Infinium 450K Human DNA methylation Beadchip	200866140110_R05C02_Grn.idat	200866140110_R05C02_Red.idat	Gene Expression Omnibus	GSE108576	GSM2905399
MBM-09	Patient with brain metastasis	Homo sapiens	Brain metastasis	Microdissected formalin-fixed paraffin-embedded tissue	Tissue specimen collection	Histopathological classification of brain metastasis	Genomic DNA extraction	Genome-wide DNA methylation profiling	Data processing and analysis	60	Male	Melanoma	NRAS mutated	2995	Dead	Illumina Infinium 450K Human DNA methylation Beadchip	7310440033_R04C01_Grn.idat	7310440033_R04C01_Red.idat	Gene Expression Omnibus	GSE44661	GSM1088546
MBM-10	Patient with brain metastasis	Homo sapiens	Brain metastasis	Microdissected formalin-fixed paraffin-embedded tissue	Tissue specimen collection	Histopathological classification of brain metastasis	Genomic DNA extraction	Genome-wide DNA methylation profiling	Data processing and analysis	63	Female	Melanoma	BRAF/NRAS wild type	5999	Dead	Illumina Infinium 450K Human DNA methylation Beadchip	7310440033_R05C01_Grn.idat	7310440033_R05C01_Red.idat	Gene Expression Omnibus	GSE44661	GSM1088547
MBM-11	Patient with brain metastasis	Homo sapiens	Brain metastasis	Microdissected formalin-fixed paraffin-embedded tissue	Tissue specimen collection	Histopathological classification of brain metastasis	Genomic DNA extraction	Genome-wide DNA methylation profiling	Data processing and analysis	80	Male	Melanoma	N/A	1461	Alive	Illumina Infinium 450K Human DNA methylation Beadchip	7786915145_R06C02_Grn.idat	7786915145_R06C02_Red.idat	Gene Expression Omnibus	GSE44661	GSM1088548
MBM-12	Patient with brain metastasis	Homo sapiens	Brain metastasis	Microdissected formalin-fixed paraffin-embedded tissue	Tissue specimen collection	Histopathological classification of brain metastasis	Genomic DNA extraction	Genome-wide DNA methylation profiling	Data processing and analysis	44	Male	Melanoma	NRAS mutated	760	Dead	Illumina Infinium 450K Human DNA methylation Beadchip	7786923108_R02C01_Grn.idat	7786923108_R02C01_Red.idat	Gene Expression Omnibus	GSE44661	GSM1088549
MBM-14	Patient with brain metastasis	Homo sapiens	Brain metastasis	Microdissected formalin-fixed paraffin-embedded tissue	Tissue specimen collection	Histopathological classification of brain metastasis	Genomic DNA extraction	Genome-wide DNA methylation profiling	Data processing and analysis	62	Male	Melanoma	BRAF mutated	943	Dead	Illumina Infinium 450K Human DNA methylation Beadchip	7786923108_R04C01_Grn.idat	7786923108_R04C01_Red.idat	Gene Expression Omnibus	GSE44661	GSM1088551
MBM-15	Patient with brain metastasis	Homo sapiens	Brain metastasis	Microdissected formalin-fixed paraffin-embedded tissue	Tissue specimen collection	Histopathological classification of brain metastasis	Genomic DNA extraction	Genome-wide DNA methylation profiling	Data processing and analysis	45	Female	Melanoma	NRAS mutated	2404	Alive	Illumina Infinium 450K Human DNA methylation Beadchip	7786923108_R05C01_Grn.idat	7786923108_R05C01_Red.idat	Gene Expression Omnibus	GSE44661	GSM1088552
MBM-16	Patient with brain metastasis	Homo sapiens	Brain metastasis	Microdissected formalin-fixed paraffin-embedded tissue	Tissue specimen collection	Histopathological classification of brain metastasis	Genomic DNA extraction	Genome-wide DNA methylation profiling	Data processing and analysis	48	Female	Melanoma	BRAF mutated	1232	Alive	Illumina Infinium 450K Human DNA methylation Beadchip	7786923108_R06C01_Grn.idat	7786923108_R06C01_Red.idat	Gene Expression Omnibus	GSE44661	GSM1088553
MBM-17	Patient with brain metastasis	Homo sapiens	Brain metastasis	Microdissected formalin-fixed paraffin-embedded tissue	Tissue specimen collection	Histopathological classification of brain metastasis	Genomic DNA extraction	Genome-wide DNA methylation profiling	Data processing and analysis	NA	Female	Melanoma	BRAF mutated	8187	Dead	Illumina Infinium 450K Human DNA methylation Beadchip	200397860098_R02C01_Grn.idat	200397860098_R02C01_Red.idat	Gene Expression Omnibus	GSE108576	GSM2905400
MBM-18	Patient with brain metastasis	Homo sapiens	Brain metastasis	Microdissected formalin-fixed paraffin-embedded tissue	Tissue specimen collection	Histopathological classification of brain metastasis	Genomic DNA extraction	Genome-wide DNA methylation profiling	Data processing and analysis	75	Female	Melanoma	NRAS mutated	3881	Dead	Illumina Infinium 450K Human DNA methylation Beadchip	200123460092_R01C01_Grn.idat	200123460092_R01C01_Red.idat	Gene Expression Omnibus	GSE108576	GSM2905401
MBM-19	Patient with brain metastasis	Homo sapiens	Brain metastasis	Microdissected formalin-fixed paraffin-embedded tissue	Tissue specimen collection	Histopathological classification of brain metastasis	Genomic DNA extraction	Genome-wide DNA methylation profiling	Data processing and analysis	56	Male	Melanoma	BRAF mutated	1185	Dead	Illumina Infinium 450K Human DNA methylation Beadchip	200397860045_R05C01_Grn.idat	200397860045_R05C01_Red.idat	Gene Expression Omnibus	GSE108576	GSM2905402
MBM-20	Patient with brain metastasis	Homo sapiens	Brain metastasis	Microdissected formalin-fixed paraffin-embedded tissue	Tissue specimen collection	Histopathological classification of brain metastasis	Genomic DNA extraction	Genome-wide DNA methylation profiling	Data processing and analysis	70	Female	Melanoma	BRAF/NRAS wild type	3710	Alive	Illumina Infinium 450K Human DNA methylation Beadchip	200397860098_R05C01_Grn.idat	200397860098_R05C01_Red.idat	Gene Expression Omnibus	GSE108576	GSM2905403
MBM-21	Patient with brain metastasis	Homo sapiens	Brain metastasis	Microdissected formalin-fixed paraffin-embedded tissue	Tissue specimen collection	Histopathological classification of brain metastasis	Genomic DNA extraction	Genome-wide DNA methylation profiling	Data processing and analysis	56	Female	Melanoma	BRAF/NRAS wild type	2014	Dead	Illumina Infinium 450K Human DNA methylation Beadchip	200866140110_R06C02_Grn.idat	200866140110_R06C02_Red.idat	Gene Expression Omnibus	GSE108576	GSM2905404
MBM-22	Patient with brain metastasis	Homo sapiens	Brain metastasis	Microdissected formalin-fixed paraffin-embedded tissue	Tissue specimen collection	Histopathological classification of brain metastasis	Genomic DNA extraction	Genome-wide DNA methylation profiling	Data processing and analysis	53	Male	Melanoma	NRAS mutated	2684	Dead	Illumina Infinium 450K Human DNA methylation Beadchip	200397540080_R05C01_Grn.idat	200397540080_R05C01_Red.idat	Gene Expression Omnibus	GSE108576	GSM2905405
MBM-23	Patient with brain metastasis	Homo sapiens	Brain metastasis	Microdissected formalin-fixed paraffin-embedded tissue	Tissue specimen collection	Histopathological classification of brain metastasis	Genomic DNA extraction	Genome-wide DNA methylation profiling	Data processing and analysis	73	Male	Melanoma	NRAS mutated	1187	Dead	Illumina Infinium 450K Human DNA methylation Beadchip	200397540080_R06C02_Grn.idat	200397540080_R06C02_Red.idat	Gene Expression Omnibus	GSE108576	GSM2905406
MBM-24	Patient with brain metastasis	Homo sapiens	Brain metastasis	Microdissected formalin-fixed paraffin-embedded tissue	Tissue specimen collection	Histopathological classification of brain metastasis	Genomic DNA extraction	Genome-wide DNA methylation profiling	Data processing and analysis	71	Male	Melanoma	NRAS mutated	2252	Dead	Illumina Infinium 450K Human DNA methylation Beadchip	200397860045_R03C01_Grn.idat	200397860045_R03C01_Red.idat	Gene Expression Omnibus	GSE108576	GSM2905407
MBM-25	Patient with brain metastasis	Homo sapiens	Brain metastasis	Microdissected formalin-fixed paraffin-embedded tissue	Tissue specimen collection	Histopathological classification of brain metastasis	Genomic DNA extraction	Genome-wide DNA methylation profiling	Data processing and analysis	51	Male	Melanoma	BRAF mutated	3541	Dead	Illumina Infinium 450K Human DNA methylation Beadchip	200397540080_R01C02_Grn.idat	200397540080_R01C02_Red.idat	Gene Expression Omnibus	GSE108576	GSM2905408
MBM-26	Patient with brain metastasis	Homo sapiens	Brain metastasis	Microdissected formalin-fixed paraffin-embedded tissue	Tissue specimen collection	Histopathological classification of brain metastasis	Genomic DNA extraction	Genome-wide DNA methylation profiling	Data processing and analysis	75	Male	Melanoma	NRAS mutated	2078	Dead	Illumina Infinium 450K Human DNA methylation Beadchip	200397860098_R03C02_Grn.idat	200397860098_R03C02_Red.idat	Gene Expression Omnibus	GSE108576	GSM2905409
MBM-27	Patient with brain metastasis	Homo sapiens	Brain metastasis	Microdissected formalin-fixed paraffin-embedded tissue	Tissue specimen collection	Histopathological classification of brain metastasis	Genomic DNA extraction	Genome-wide DNA methylation profiling	Data processing and analysis	82	Male	Melanoma	BRAF mutated	4217	Dead	Illumina Infinium 450K Human DNA methylation Beadchip	200397540080_R04C02_Grn.idat	200397540080_R04C02_Red.idat	Gene Expression Omnibus	GSE108576	GSM2905410
MBM-28	Patient with brain metastasis	Homo sapiens	Brain metastasis	Microdissected formalin-fixed paraffin-embedded tissue	Tissue specimen collection	Histopathological classification of brain metastasis	Genomic DNA extraction	Genome-wide DNA methylation profiling	Data processing and analysis	47	Male	Melanoma	BRAF mutated	4867	Alive	Illumina Infinium 450K Human DNA methylation Beadchip	200397540080_R03C02_Grn.idat	200397540080_R03C02_Red.idat	Gene Expression Omnibus	GSE108576	GSM2905411
MBM-29	Patient with brain metastasis	Homo sapiens	Brain metastasis	Microdissected formalin-fixed paraffin-embedded tissue	Tissue specimen collection	Histopathological classification of brain metastasis	Genomic DNA extraction	Genome-wide DNA methylation profiling	Data processing and analysis	74	Male	Melanoma	NRAS mutated	209	Dead	Illumina Infinium 450K Human DNA methylation Beadchip	201031670094_R01C02_Grn.idat	201031670094_R01C02_Red.idat	Gene Expression Omnibus	GSE108576	GSM2905412
MBM-30	Patient with brain metastasis	Homo sapiens	Brain metastasis	Microdissected formalin-fixed paraffin-embedded tissue	Tissue specimen collection	Histopathological classification of brain metastasis	Genomic DNA extraction	Genome-wide DNA methylation profiling	Data processing and analysis	64	Male	Melanoma	BRAF/NRAS wild type	1296	Alive	Illumina Infinium 450K Human DNA methylation Beadchip	201031670094_R02C02_Grn.idat	201031670094_R02C02_Red.idat	Gene Expression Omnibus	GSE108576	GSM2905413
MBM-31	Patient with brain metastasis	Homo sapiens	Brain metastasis	Microdissected formalin-fixed paraffin-embedded tissue	Tissue specimen collection	Histopathological classification of brain metastasis	Genomic DNA extraction	Genome-wide DNA methylation profiling	Data processing and analysis	72	Male	Melanoma	NRAS mutated	2465	Dead	Illumina Infinium 450K Human DNA methylation Beadchip	200397860045_R04C01_Grn.idat	200397860045_R04C01_Red.idat	Gene Expression Omnibus	GSE108576	GSM2905414
MBM-32	Patient with brain metastasis	Homo sapiens	Brain metastasis	Microdissected formalin-fixed paraffin-embedded tissue	Tissue specimen collection	Histopathological classification of brain metastasis	Genomic DNA extraction	Genome-wide DNA methylation profiling	Data processing and analysis	46	Female	Melanoma	BRAF mutated	3207	Dead	Illumina Infinium 450K Human DNA methylation Beadchip	200397540080_R02C02_Grn.idat	200397540080_R02C02_Red.idat	Gene Expression Omnibus	GSE108576	GSM2905415
MBM-33	Patient with brain metastasis	Homo sapiens	Brain metastasis	Microdissected formalin-fixed paraffin-embedded tissue	Tissue specimen collection	Histopathological classification of brain metastasis	Genomic DNA extraction	Genome-wide DNA methylation profiling	Data processing and analysis	53	Male	Melanoma	BRAF mutated	654	Dead	Illumina Infinium 450K Human DNA methylation Beadchip	200397860045_R01C02_Grn.idat	200397860045_R01C02_Red.idat	Gene Expression Omnibus	GSE108576	GSM2905416
MBM-34	Patient with brain metastasis	Homo sapiens	Brain metastasis	Microdissected formalin-fixed paraffin-embedded tissue	Tissue specimen collection	Histopathological classification of brain metastasis	Genomic DNA extraction	Genome-wide DNA methylation profiling	Data processing and analysis	55	Male	Melanoma	NRAS mutated	1152	Dead	Illumina Infinium 450K Human DNA methylation Beadchip	200397860045_R06C01_Grn.idat	200397860045_R06C01_Red.idat	Gene Expression Omnibus	GSE108576	GSM2905417
MBM-35	Patient with brain metastasis	Homo sapiens	Brain metastasis	Microdissected formalin-fixed paraffin-embedded tissue	Tissue specimen collection	Histopathological classification of brain metastasis	Genomic DNA extraction	Genome-wide DNA methylation profiling	Data processing and analysis	62	Male	Melanoma	BRAF mutated	1000	Dead	Illumina Infinium 450K Human DNA methylation Beadchip	200397540080_R01C01_Grn.idat	200397540080_R01C01_Red.idat	Gene Expression Omnibus	GSE108576	GSM2905418
MBM-36	Patient with brain metastasis	Homo sapiens	Brain metastasis	Microdissected formalin-fixed paraffin-embedded tissue	Tissue specimen collection	Histopathological classification of brain metastasis	Genomic DNA extraction	Genome-wide DNA methylation profiling	Data processing and analysis	40	Male	Melanoma	BRAF mutated	6684	Alive	Illumina Infinium 450K Human DNA methylation Beadchip	200397540080_R03C01_Grn.idat	200397540080_R03C01_Red.idat	Gene Expression Omnibus	GSE108576	GSM2905419
MBM-37	Patient with brain metastasis	Homo sapiens	Brain metastasis	Microdissected formalin-fixed paraffin-embedded tissue	Tissue specimen collection	Histopathological classification of brain metastasis	Genomic DNA extraction	Genome-wide DNA methylation profiling	Data processing and analysis	58	Male	Melanoma	NRAS mutated	1383	Dead	Illumina Infinium 450K Human DNA methylation Beadchip	200397540080_R06C01_Grn.idat	200397540080_R06C01_Red.idat	Gene Expression Omnibus	GSE108576	GSM2905420
MBM-38	Patient with brain metastasis	Homo sapiens	Brain metastasis	Microdissected formalin-fixed paraffin-embedded tissue	Tissue specimen collection	Histopathological classification of brain metastasis	Genomic DNA extraction	Genome-wide DNA methylation profiling	Data processing and analysis	49	Female	Melanoma	BRAF mutated	6658	Alive	Illumina Infinium 450K Human DNA methylation Beadchip	200397540080_R04C01_Grn.idat	200397540080_R04C01_Red.idat	Gene Expression Omnibus	GSE108576	GSM2905421
MBM-39	Patient with brain metastasis	Homo sapiens	Brain metastasis	Microdissected formalin-fixed paraffin-embedded tissue	Tissue specimen collection	Histopathological classification of brain metastasis	Genomic DNA extraction	Genome-wide DNA methylation profiling	Data processing and analysis	64	Male	Melanoma	BRAF mutated	1713	Dead	Illumina Infinium 450K Human DNA methylation Beadchip	9305216131_R06C02_Grn.idat	9305216131_R06C02_Red.idat	Gene Expression Omnibus	GSE108576	GSM2905422
MBM-40	Patient with brain metastasis	Homo sapiens	Brain metastasis	Microdissected formalin-fixed paraffin-embedded tissue	Tissue specimen collection	Histopathological classification of brain metastasis	Genomic DNA extraction	Genome-wide DNA methylation profiling	Data processing and analysis	55	Male	Melanoma	NRAS mutated	555	Dead	Illumina Infinium 450K Human DNA methylation Beadchip	200397540080_R02C01_Grn.idat	200397540080_R02C01_Red.idat	Gene Expression Omnibus	GSE108576	GSM2905423
MBM-41	Patient with brain metastasis	Homo sapiens	Brain metastasis	Microdissected formalin-fixed paraffin-embedded tissue	Tissue specimen collection	Histopathological classification of brain metastasis	Genomic DNA extraction	Genome-wide DNA methylation profiling	Data processing and analysis	62	Female	Melanoma	NRAS mutated	119	Dead	Illumina Infinium 450K Human DNA methylation Beadchip	200397540080_R05C02_Grn.idat	200397540080_R05C02_Red.idat	Gene Expression Omnibus	GSE108576	GSM2905424
MBM-42	Patient with brain metastasis	Homo sapiens	Brain metastasis	Microdissected formalin-fixed paraffin-embedded tissue	Tissue specimen collection	Histopathological classification of brain metastasis	Genomic DNA extraction	Genome-wide DNA methylation profiling	Data processing and analysis	32	Male	Melanoma	BRAF mutated	5648	Dead	Illumina Infinium 450K Human DNA methylation Beadchip	9305216131_R05C02_Grn.idat	9305216131_R05C02_Red.idat	Gene Expression Omnibus	GSE108576	GSM2905425
MBM-43	Patient with brain metastasis	Homo sapiens	Brain metastasis	Microdissected formalin-fixed paraffin-embedded tissue	Tissue specimen collection	Histopathological classification of brain metastasis	Genomic DNA extraction	Genome-wide DNA methylation profiling	Data processing and analysis	48	Male	Melanoma	NRAS mutated	1895	Dead	Illumina Infinium 450K Human DNA methylation Beadchip	200397860098_R05C02_Grn.idat	200397860098_R05C02_Red.idat	Gene Expression Omnibus	GSE108576	GSM2905426
MBM-44	Patient with brain metastasis	Homo sapiens	Brain metastasis	Microdissected formalin-fixed paraffin-embedded tissue	Tissue specimen collection	Histopathological classification of brain metastasis	Genomic DNA extraction	Genome-wide DNA methylation profiling	Data processing and analysis	72	Male	Melanoma	BRAF mutated	1810	Dead	Illumina Infinium 450K Human DNA methylation Beadchip	9305216131_R04C02_Grn.idat	9305216131_R04C02_Red.idat	Gene Expression Omnibus	GSE108576	GSM2905427
MBM-45	Patient with brain metastasis	Homo sapiens	Brain metastasis	Microdissected formalin-fixed paraffin-embedded tissue	Tissue specimen collection	Histopathological classification of brain metastasis	Genomic DNA extraction	Genome-wide DNA methylation profiling	Data processing and analysis	42	Female	Melanoma	BRAF mutated	919	Dead	Illumina Infinium 450K Human DNA methylation Beadchip	200397860045_R02C01_Grn.idat	200397860045_R02C01_Red.idat	Gene Expression Omnibus	GSE108576	GSM2905428
MBM-46	Patient with brain metastasis	Homo sapiens	Brain metastasis	Microdissected formalin-fixed paraffin-embedded tissue	Tissue specimen collection	Histopathological classification of brain metastasis	Genomic DNA extraction	Genome-wide DNA methylation profiling	Data processing and analysis	80	Male	Melanoma	BRAF mutated	1716	Dead	Illumina Infinium 450K Human DNA methylation Beadchip	8784241079_R04C01_Grn.idat	8784241079_R04C01_Red.idat	Gene Expression Omnibus	GSE108576	GSM2905429
MBM-47	Patient with brain metastasis	Homo sapiens	Brain metastasis	Microdissected formalin-fixed paraffin-embedded tissue	Tissue specimen collection	Histopathological classification of brain metastasis	Genomic DNA extraction	Genome-wide DNA methylation profiling	Data processing and analysis	59	Male	Melanoma	BRAF mutated	1271	Dead	Illumina Infinium 450K Human DNA methylation Beadchip	7800246021_R03C01_Grn.idat	7800246021_R03C01_Red.idat	Gene Expression Omnibus	GSE108576	GSM2905430
MBM-48	Patient with brain metastasis	Homo sapiens	Brain metastasis	Microdissected formalin-fixed paraffin-embedded tissue	Tissue specimen collection	Histopathological classification of brain metastasis	Genomic DNA extraction	Genome-wide DNA methylation profiling	Data processing and analysis	56	Female	Melanoma	NRAS mutated	3471	Dead	Illumina Infinium 450K Human DNA methylation Beadchip	7310440033_R03C01_Grn.idat	7310440033_R03C01_Red.idat	Gene Expression Omnibus	GSE108576	GSM2905431
MBM-49	Patient with brain metastasis	Homo sapiens	Brain metastasis	Microdissected formalin-fixed paraffin-embedded tissue	Tissue specimen collection	Histopathological classification of brain metastasis	Genomic DNA extraction	Genome-wide DNA methylation profiling	Data processing and analysis	54	Male	Melanoma	NRAS mutated	1926	Dead	Illumina Infinium 450K Human DNA methylation Beadchip	7800246020_R01C01_Grn.idat	7800246020_R01C01_Red.idat	Gene Expression Omnibus	GSE108576	GSM2905432
MBM-50	Patient with brain metastasis	Homo sapiens	Brain metastasis	Microdissected formalin-fixed paraffin-embedded tissue	Tissue specimen collection	Histopathological classification of brain metastasis	Genomic DNA extraction	Genome-wide DNA methylation profiling	Data processing and analysis	44	Male	Melanoma	BRAF mutated	1688	Dead	Illumina Infinium 450K Human DNA methylation Beadchip	200397860045_R01C01_Grn.idat	200397860045_R01C01_Red.idat	Gene Expression Omnibus	GSE108576	GSM2905433
MBM-51	Patient with brain metastasis	Homo sapiens	Brain metastasis	Microdissected formalin-fixed paraffin-embedded tissue	Tissue specimen collection	Histopathological classification of brain metastasis	Genomic DNA extraction	Genome-wide DNA methylation profiling	Data processing and analysis	77	Male	Melanoma	N/A	514	Dead	Illumina Infinium 450K Human DNA methylation Beadchip	8784241079_R03C01_Grn.idat	8784241079_R03C01_Red.idat	Gene Expression Omnibus	GSE108576	GSM2905434
MBM-52	Patient with brain metastasis	Homo sapiens	Brain metastasis	Microdissected formalin-fixed paraffin-embedded tissue	Tissue specimen collection	Histopathological classification of brain metastasis	Genomic DNA extraction	Genome-wide DNA methylation profiling	Data processing and analysis	73	Male	Melanoma	BRAF/NRAS wild type	904	Dead	Illumina Infinium 450K Human DNA methylation Beadchip	7800246021_R01C01_Grn.idat	7800246021_R01C01_Red.idat	Gene Expression Omnibus	GSE108576	GSM2905435
MBM-53	Patient with brain metastasis	Homo sapiens	Brain metastasis	Microdissected formalin-fixed paraffin-embedded tissue	Tissue specimen collection	Histopathological classification of brain metastasis	Genomic DNA extraction	Genome-wide DNA methylation profiling	Data processing and analysis	54	Male	Melanoma	NRAS mutated	3719	Dead	Illumina Infinium 450K Human DNA methylation Beadchip	7800246021_R02C01_Grn.idat	7800246021_R02C01_Red.idat	Gene Expression Omnibus	GSE108576	GSM2905436
